# Review of the planthopper genus *Neodurium* Fennah, 1956 (Hemiptera, Fulgoromorpha, Issidae)

**DOI:** 10.3897/zookeys.517.8817

**Published:** 2015-08-12

**Authors:** Zhi-Min Chang, Xiang-Sheng Chen, Mick Webb

**Affiliations:** 1 Institute of Entomology / Special Key Laboratory for Development and Utilization of Insect Resources, Guizhou University, Guiyang, Guizhou, 550025, P.R. China; 2 The Provincial Key Laboratory for Agricultural Pest Management of Mountainous Regions, Guizhou University, Guiyang, Guizhou, 550025, P.R. China; 3 Department of Entomology, The Natural History Museum, Cromwell Road, South Kensington, London, U.K.

**Keywords:** Fulgoroidea, planthopper, Oriental region, female genitalia, new species

## Abstract

The planthopper genus *Neodurium* Fennah is reviewed and *Neodurium fennahi* Chang & Chen, **sp. n.** (Hemiptera: Fulgoromorpha: Issidae) from China (Yunnan), is described and illustrated. A checklist of the species of *Neodurium* is given and a key provided for their separation. The female genitalia of *Neodurium* species are described for the first time.

## Introduction

The Chinese planthopper genus *Neodurium* (Issidae) was established by Fennah (1956) for *Neodurium postfasciatum* Fennah based on a female specimen from Hubei in China. Later, Ran et al. (2005) added information on the male genitalia for this species and described two more species. Subsequently, Zhang and Chen (2008) placed the genus in the Issidae, tribe Parahiraciini Cheng & Yang, and added further two new species. Then Wang and Wang (2011) described another new species. Accordingly the genus *Neodurium* is endemic to China and currently comprises seven species including the species described below. The tribe Parahiraciini is endemic to Indo-Malayan Realm with few taxa distributed also in the Eastern Palaearctic Subrealm and comprises 14 genera (Gnezdilov 2013).

Herein, a further new species of *Neodurium* from China is described and illustrated, and all other species are reviewed. A checklist to the species of *Neodurium* is given together with a key for their separation. The female genitalia of the genus is described for the first time.

## Material and menthods

The morphological terminology of the head and body follows Chan and Yang (1994), and the terminology of male and female genitalia follows Gnezdilov (2002) and Gnezdilov (2003). Dry specimens were used for descriptions and illustrations. External morphology was observed under a stereoscopic microscope. Measurements are given in millimeters. The genital segments of the examined specimens were macerated in 10% KOH, washed in water and transferred to glycerine. Illustrations of the specimens were made with a Leica MZ 12.5 stereomicroscope. Photographs were taken with a KEYENCE VHX-1000C.

The type specimens and other specimens are deposited in the following institutions whose names are abbreviated in the text as follows:

IEGU The Institute of Entomology, Guizhou University, Guiyang, China.

BMNH The Natural History Museum, London, UK.

IZCAS Zoological Museum, Institute of Zoology, Chinese Academy of Sciences, Beijing, China.

NWAFU The Entomological Museum, Northwest A & F University, Xi’an, China.

CAAS The California Academy of Sciences, San Francisco, USA.

## Taxonomy

### Genus Neodurium

Taxon classificationAnimaliaHemipteraIssidae

Fennah, 1956

Neodurium Fennah, 1956: 511; Ran et al. 2005: 570; Zhang and Chen 2008: 64; Wang and Wang 2011: 551.

#### Type species.

*Neodurium postfasciatum* Fennah, 1956

#### Diagnosis.

Small-sized issids. Vertex pentagonal, anterior margin obtuse-angled convex, posterior angulately excavate, marginal carinae straight. Pronotum as long as vertex, with small pit between median carina and lateral carina. Mesonotum shorter than vertex and pronotum together, with median cargin obscure. Forewings oval, with claval suture absent or obscure, M simple. Hind wings incised on the margin into two lobes, anal lobe absent or excessively reduced, veins simple. Fore- and mesofemora flattened; hind tibia with a small sub-basal spine and two distinct lateral spines.

#### Checklist of species of *Neodurium* Fennah

*Neodurium digitiformum* Ran & Liang, 2005: 571: figs 9–16; China (Hubei)

*Neodurium duplicadigitum* Zhang & Chen, 2008: 66: figs 10–18; China (Yunnan)

*Neodurium fennahi* Chang & Chen, sp. n.: figs 2–3; China (Yunnan)

*Neodurium flatidum* Ran & Liang, 2005: 572: figs 17–24; China (Yunnan)

*Neodurium hamatum* Wang & Wang, 2011: 553: figs 1–24; China (Yunnan)

*Neodurium postfasciatum* Fennah, 1956: 513: figs 24E–I; China (Hubei, Sichuan, Yunnan)

*Neodurium weiningensis* Zhang & Chen, 2008: 65: figs 1–9; China (Guizhou)

#### Key to species of *Neodurium* (males)

**Table:** 

1	Anal tube without lobes (Figs 5A); genital styles without triangular process at midlength of dorsal margin in lateral view (Fig. 5D)	***Neodurium hamatum* Wang & Wang**
–	Anal tube with sub-basal angular lobes in dorsal view (Figs 2G, 4A, 6A, 7A); genital styles with triangular process in midlength of dorsal margin in lateral view (Fig. 2F: c)	**2**
2	Phallobase without any process on dorsal surface (see Ran et al. 2005: fig. 23)	***Neodurium flatidum* Ran & Liang**
–	Phallobase with a process on dorsal surface (Figs 2J–I, 4C, 6C, 7C)	**3**
3	Anal tube subquadrate, truncate in apical margin (Fig. 4A); ventrally with “V”-shape process near middle (Fig. 4B)	***Neodurium duplicadigitum* Zhang & Chen**
–	Anal tube not truncate, with several lobes in apical margin	**4**
4	Phallobase with small finger-like process (see Ran et al. 2005: Fig. 15)	***Neodurium digitiformum* Ran & Liang**
–	Phallobase with fan-like or strap-shaped process (Figs 2J, 6C, 7C)	**5**
5	Phallobase with two pairs of strap-shaped processes (Fig. 2J), one pair of processes connected medially (Fig. 2I)	***Neodurium fennahi* Chang & Chen, sp. n.**
–	Phallobase with fan-like process (Fig. 6C)	**6**
6	Phallobase with small dentate tooth near fan-like process on dorsal margin; aedeagus with pair of long hooks (Fig. 6C)	***Neodurium postfasciatum* Fennah**
–	Phallobase without teeth near fan-like process on dorsal margin; aedeagus with a pair of short hooks (Fig. 7C)	***Neodurium weiningensis* Zhang & Chen**

#### Key to species of *Neodurium* (females)

Note: Females of *Neodurium digitiformum*, *Neodurium flatidum* and *Neodurium postfasciatum* are unknown.

**Table:** 

1	Anal tube pear-shaped with basal half broad (Fig. 3D)	***Neodurium fennahi* Chang & Chen, sp. n.**
–	Anal tube of another shape (Figs 4D, 5E, 7D)	**2**
2	Posterior connective lamina of gonapophyse VIII with ventral posterior lobes bent at broad angle, apical part hook-like (Fig. 5H: a); median field with dorsomedial process club-like (Fig. 5H: b)	***Neodurium hamatum* Wang & Wang**
–	Posterior connective lamina of gonapophyse VIII with ventral posterior lobes bent at slender angle, blade-like; median field with dorsomedial process broad	**3**
3	Posterior connective lamina with dorsomedial processes arcuate (Fig. 4G: b)	***Neodurium duplicadigitum* Zhang & Chen**
–	Posterior connective lamina with dorsomedial process sub-quadrate (Fig. 7G: b)	***Neodurium weiningensis* Zhang & Chen**

### Neodurium fennahi

Taxon classificationAnimaliaHemipteraIssidae

Chang & Chen sp. n.

http://zoobank.org/6189B6EB-E070-4893-BBD8-0A46DAD90FE8

Figs 1E , 2 , 3

#### Type material.

Holotype: ♂, China: Guizhou, Yuxi, Ailao Mountain National Nature Reserve (24°12'N, 101°19'E), 21 July 2011, S.-Y. Xu, W.-B. Zheng and W.-C. Yang (IEGU); paratypes: 11♂♂, 6♀♀, same data as holotype (IEGU); 1♂, 1♀, same data as holotype (BMNH).

#### Description.

Body length (from apex of vertex to tip of forewing): male 6.0–6.2 mm, female 6.8–7.0 mm; Forewing: male 5.0–5.2 mm, female 5.6–5.8 mm.

**Figures 1.**
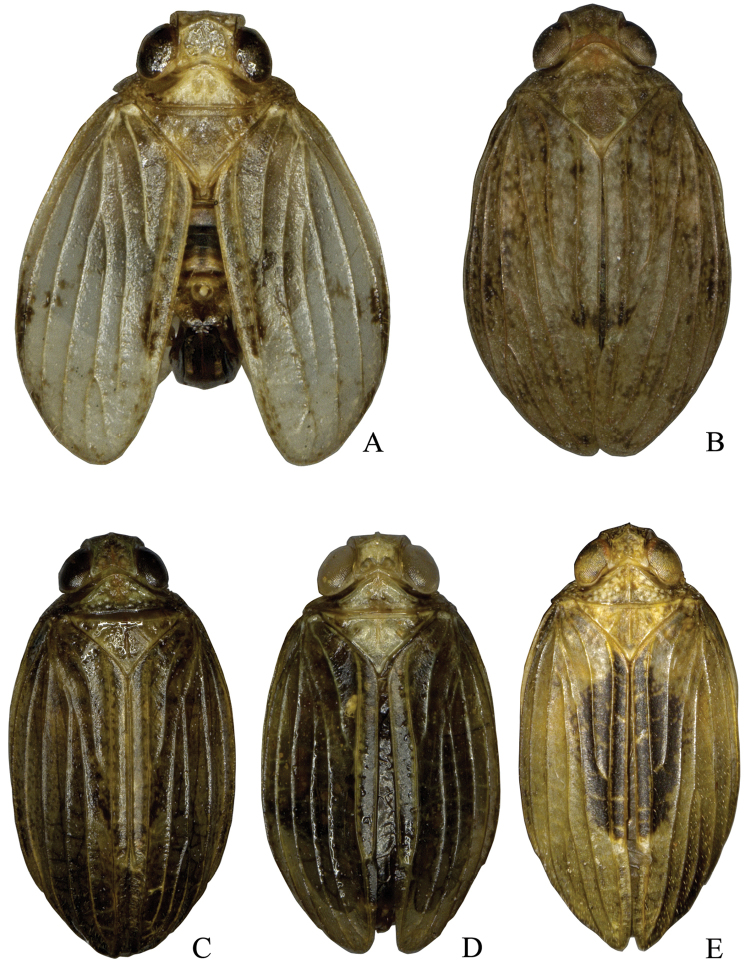
Dorsal habitus of *Neodurium* species. **A** *Neodurium duplicadigitum* Zhang & Chen, 2008 **B** *Neodurium hamatum* Wang & Wang, 2011 **C** *Neodurium postfasciatum* Fennah, 1956 **D** *Neodurium weiningensis* Zhang & Chen, 2008 **E** *Neodurium fennahi* Chang & Chen, sp. n.

**Figure 2.**
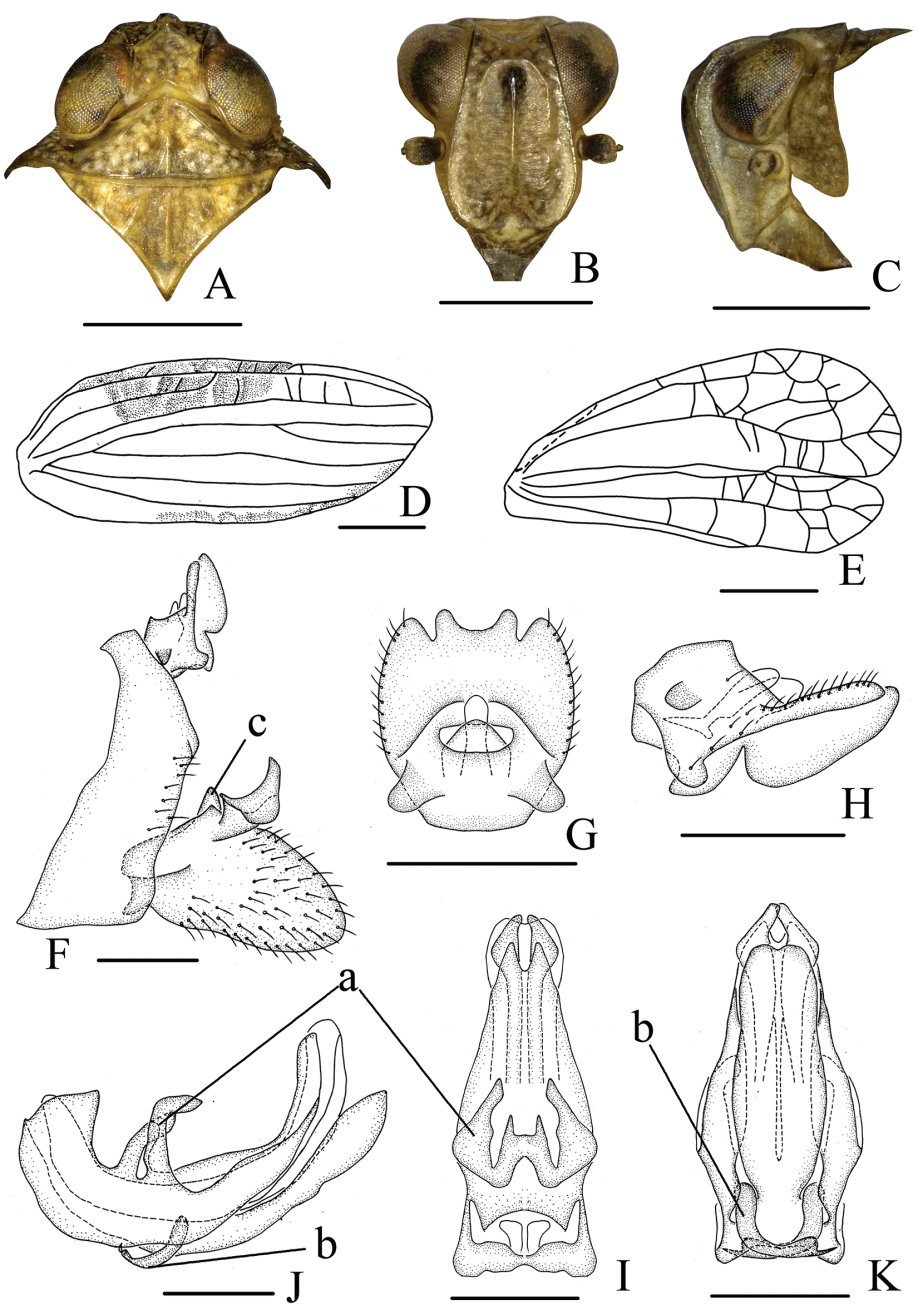
*Neodurium fennahi* Chang & Chen, sp. n., male **A** head and thorax, dorsal view **B** head, ventral view **C** head and thorax, lateral view **D** forewing **E** hind wing **F** male genitalia, lateral view **G** anal segment, dorsal view **H** anal segment, lateral view **I** aedeagus and phallobase, dorsal view **J** aedeagus and phallobase, lateral view **K** aedeagus and phallobase, ventral view. a- triangular process, b- strap-shaped processes, c- hook-like processes. Scale bars: 1.0 mm (**A–E**); 0.5 mm (**F–K**).

**Coloration.** General colour (Figs 1E, 2A–C) brown with pale mottling on the vertex and pronotum and base of frons. Eyes reddish brown to dark brown; antenna dark brown; frons (Fig. 2B) with dark brown spot dorsomedially; clypeus brown to dark brown; rostrum dark brown. Forewings (Fig. 1E) with dark semicircular mark on clavus forming ovoid patch with wings at rest. Legs with tips of spines on hind tibiae and tarsi black.

**Head and thorax.** Head (Fig. 2A) including eyes narrower than pronotum (0.74: 1.00). Vertex (Fig. 2A) shorter in middle than the wide at base (0.70: 1.00), strongly dorsoventrally depressed; disc of vertex with one obscure median carina. Frons (Fig. 2B) flat, disc slightly depressed, basal margin arched in aute angle, apical margin obtusely rounded, lateral margin ridged, lateral margins of frons incurved below level of socket of antennae, longer in middle than the widest breath (1.00: 0.78), with median carina and lateral carina, lateral carina reaching to the level of antennae. Pronotum (Fig. 2B) with median carina and lateral carina, lateral carina not reaching to the posterior margin. Mesonotum (Fig. 2A) triangular, with median carina and lateral carina. Hind tibiae each with one small spine near base and two distinct lateral spines, spinal formula of hind leg 8-14-2. Forewings (Fig. 2D) long, subquadrate, 2.8 times as long as maximum width; longitudinal veins distinct, Sc+R vein long, reaching beyond half length of tegmen, Sc and R seperated near base, M vein divided into three branches, CuA vein (cubitus anterior) not forked, claval vein Pcu (postcubitus) and A_1_ veins uniting in middle of tegmina. Hind wings (Fig. 2E) incised on apical margin into two lobes, anal lobe reduced, reticulate apically.

**Male genitalia.** Anal tube (Fig. 2G) relatively short, subquadrate in dorsal view, with two lobes near basal part, with four blunt lobes in apical margin. Anal column (Fig. 2G) short, located at the middle of anal tube. Pygofer (Fig. 2E) irregular subquadrate in lateral view, anterior margin moderately concave, posterior margin bended to ventro-lateral side. Genital styles (Fig. 2E) moderately long, dorsal margin producing a triangular inward lobe near capitulum; capitulum of style narrowing apically on short neck. Phallobase with dorsal lobe relatively long, not reaching the tip of lateral lobe; with two pairs of strap-shaped processes (dorsal process) near base in lateral view (Fig. 2J) and one pair of processes intermediately connected, forming “H”- shaped bridge in dorsal view (Fig. 2I); ventral lobe long, apex weakly sinuate in ventral view (Fig. 2K); lateral lobe split into two branches in ventral view (Fig. 2K). Aedeagus with pair of long convergent hook-like processes (Fig. 2I).

**Figure 3.**
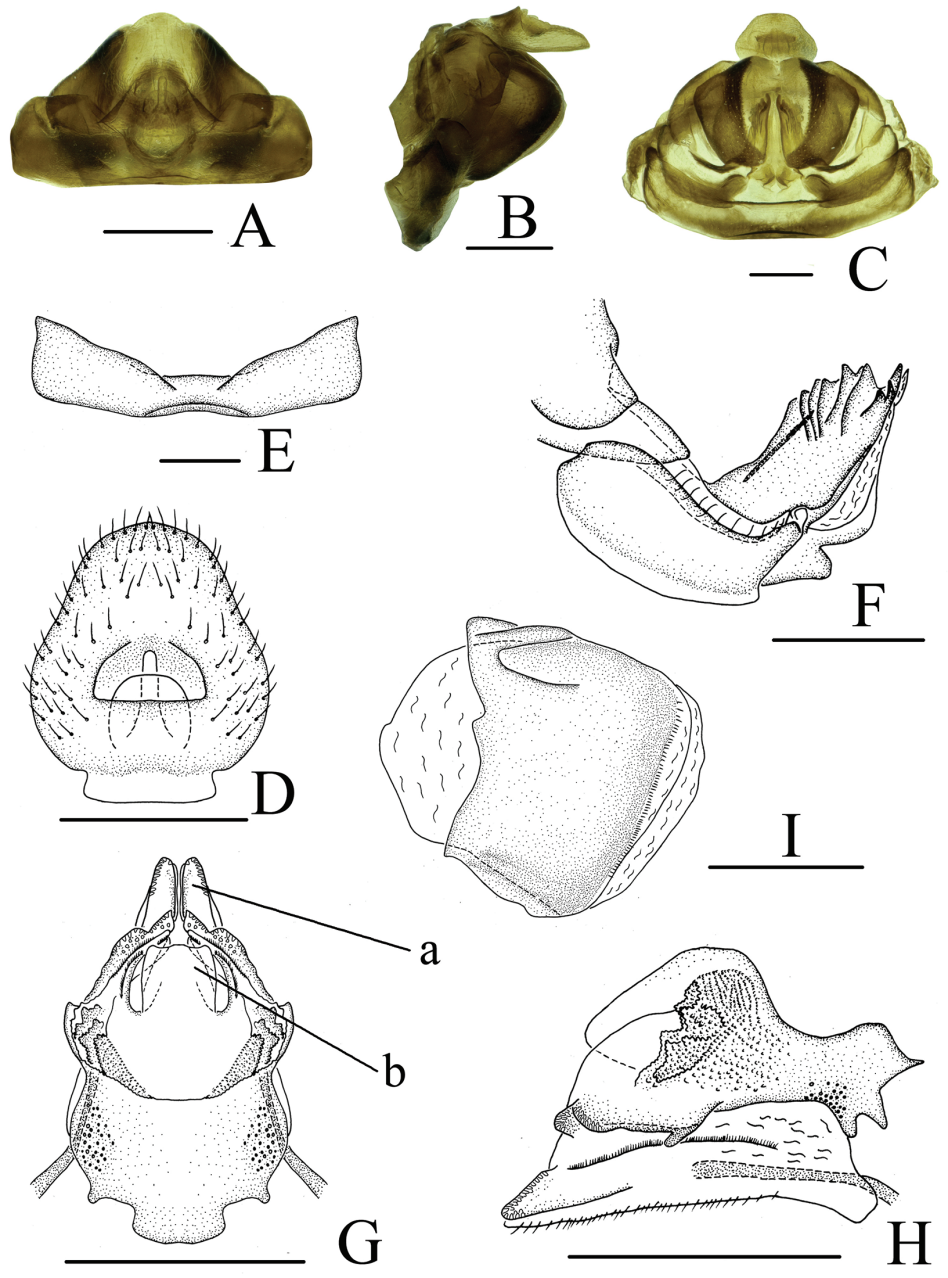
*Neodurium triprocessum* Chang & Chen, sp. n., female **A** female genitalia, dorsal view **B** female genitalia, lateral view **C** female genitalia, ventral view **D** anal segment, dorsal view **E** sternum VII, ventral view **F** anterior connective lamina of gonapophyses VIII, lateral view **G** posterior connective lamina of gonapophyses IX, dorsal view **H** posterior connective lamina of gonapophyses IX, lateral view **I** gonoplacs, lateral view. a- posterior ventral lobes, b- process of median field. Scale bars: 0.5 mm (**A–I**).

**Female genitalia.** Hind margin of sternum VII with deep and wide median concavity (Figs 3C, 3E) in ventral view. Anal tube (Fig. 3D) pear-shaped with basal 1/2 broader; anal column short, located at basal 1/3 of anal tube. Hind margin of gonocoxa VIII lobe-shaped in proximal part, endogonocoxal lobe relative broad; endogonocoxal process gradually narrowing (Fig. 3F). Anterior connective lamina of gonapophyses VIII (Fig. 3F) with 5 teeth bearing 5 keels in lateral group and 3 teeth in apical group; lateral fields of posterior connective lamina of gonapophyses IX (Fig. 3G, H) broad, with one obtuse and lamellar process on lateral margins; with scaly bulges between lateral margin and median field; median field with a subquadrate prominence (medial dorsal process) (Fig. 3G: b); ventroposterior lobes bent at obtuse angle (posterior ventral lobes) (Fig. 3G: a). Gonoplacs (Fig. 3I) without keels.

#### Etymology.

The new species is named for the memory of R.G. Fennah, who established the genus *Neodurium*.

#### Distribution.

China (Yunnan).

#### Remarks.

This species can be distinguished from other congeners by the dark oval patch on the forewings (Fig. 1E), phallobase with dorsal lobe (Fig. 2J) with two pairs of strap-shaped processes in lateral view (Fig. 2I).

### Neodurium digitiformum

Taxon classificationAnimaliaHemipteraIssidae

Ran & Liang, 2005

Neodurium digitiformum Ran & Liang, 2005: 571: figs 9–16; Wang and Wang 2011: 552.

#### Material examined.

No specimens have been studied by the authors.

#### Remarks.

This species was described after the holotype [1 male] from China (Hubei), deposited in IZCAS.

### Neodurium duplicadigitum

Taxon classificationAnimaliaHemipteraIssidae

Zhang & Chen, 2008

Figs 1A , 4

Neodurium duplicadigitum Zhang & Chen, 2008: 66: figs 10–18.

#### Material examined.

2 ♂♂, 1 ♀ China, Yunnan, Dali, 4 Aug. 2006, Z.-G. Zhang (IEGU).

#### Female genitalia.

**Figure 4.**
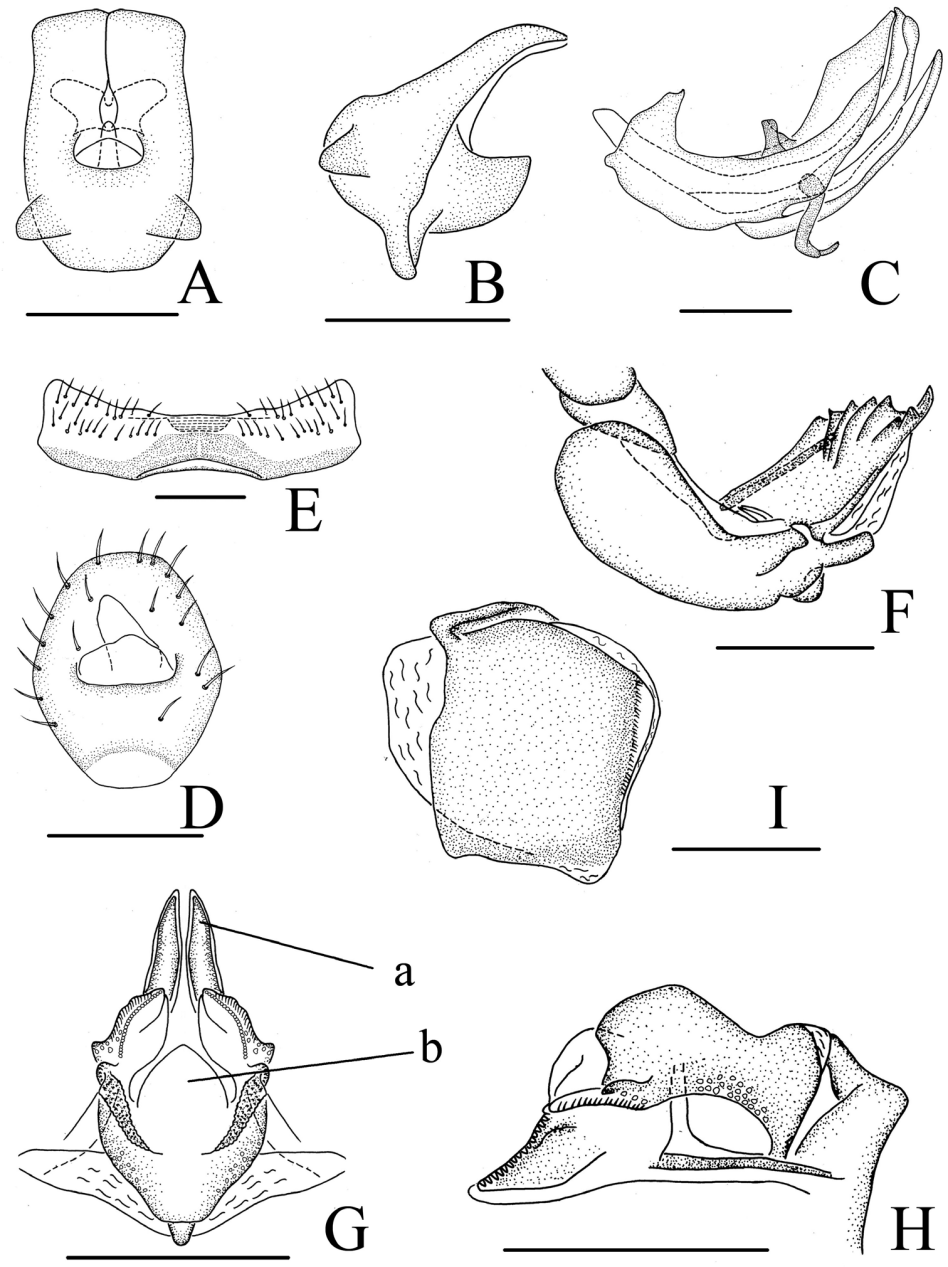
*Neodurium duplicadigitum* Zhang & Chen, 2008. **A–C** male **A** anal segment, dorsal view **B** anal segment, lateral view **C** aedeagus and phallobase, lateral view **D–I** female **D** anal segment, dorsal view **E** sternum VII, ventral view **F** anterior connective lamina of gonapophyses VIII, lateral view **G** posterior connective lamina of gonapophyses IX, dorsal view **H** posterior connective lamina of gonapophyses IX, lateral view **I** gonoplacs, lateral view. a- posterior ventral lobes, b- process of median field. Scale bars: 0.5 mm (**A–I**).

As in *Neodurium fennahi* but hind margin of sternum VII (Fig. 4E) with more wider median concavity in ventral view. Anal tube (Fig. 4D) approximately oval, truncate apically, the widest breadth in the middle; anal colum located in the middle of anal tube. Anterior connective lamina of gonapophyses VIII (Fig. 4F) with 4 teeth bearing 4 keels in lateral group. Posterior connective lamina of gonapophyses (Fig. 4G: b) IX with median field with a semicircular prominence (medial dorsal process); distal parts of posterior ventral lobes bent at slender angle, blade-like (Fig. 4G: a).

#### Remarks.

This species was described from the holotype and paratypes from China (Yunnan), deposited in IEGU.

### Neodurium flatidum

Taxon classificationAnimaliaHemipteraIssidae

Ran & Liang, 2005

Neodurium flatidum Ran & Liang, 2005: 572: figs 17–24.

#### Material examined.

No specimens have been studied by the authors.

#### Remarks.

This species was described after the holotype [1 male] from China (Yunnan), deposited in IZCAS.

### Neodurium hamatum

Taxon classificationAnimaliaHemipteraIssidae

Wang & Wang, 2011

Figs 1B , 5

Neodurium hamatum Wang & Wang, 2011: 552: figs 17–24.

#### Material examined.

1♂1♀, China, Yunnan, Ruili, 6~7 June 2011, J.-K. Long (IEGU); 1♂, Yunnan, Yingjiang, 1 June 2011, J.-K. Long (IEGU); 2♂♂, Yunnan, Ruili, 17 July 2013, W.-C. Yang (IEGU); 1♀, Yunnan, Gaoligong Mountain, Baihualing, 8 May 2009, Z.-H. Yang (IEGU).

#### Female genitalia.

**Figure 5.**
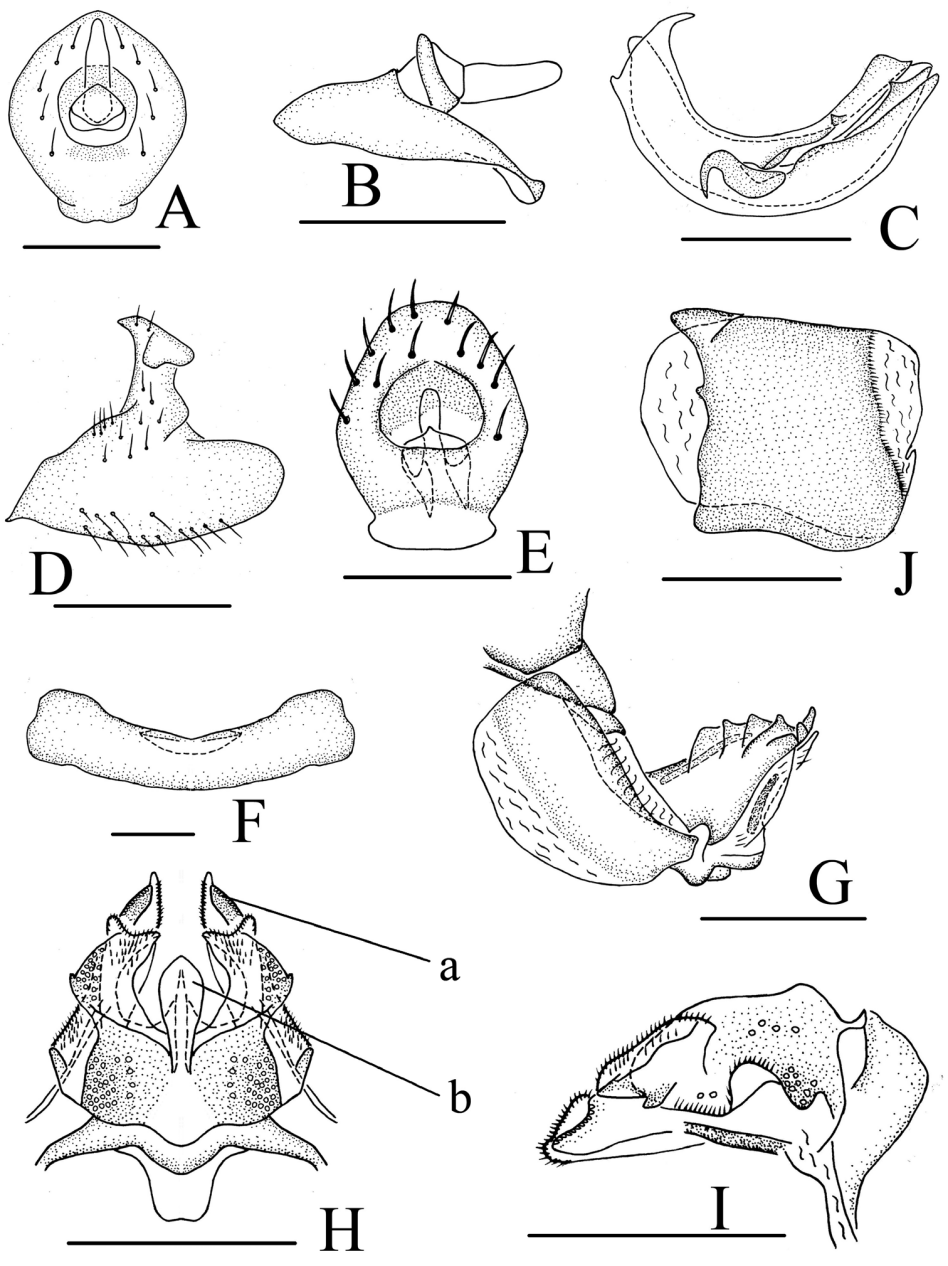
*Neodurium hamatum* Wang & Wang, 2011. **A–D** male **A** anal segment, dorsal view **B** anal segment, lateral view **C** aedeagus and phallobase, lateral view **D** genital styles, lateral view **E–J** female **E** anal segment, dorsal view **F** sternum VII, ventral view **G** anterior connective lamina of gonapophyses VIII, lateral view **H** posterior connective lamina of gonapophyses IX, dorsal view **I** posterior connective lamina of gonapophyses IX, lateral view **J** gonoplacs, lateral view. a- posterior ventral lobes, b- process of median field. Scale bars: 0.5 mm (**A–J**).

As in *Neodurium fennahi* but hind margin of sternum VII (Fig. 5F) with wide median concavity in ventral view. Anal tube (Fig. 5E) approximately oval, truncate apically, the widest breadth in the middle; anal colum located in the middle of anal tube. Anterior connective lamina of gonapophyses VIII (Fig. 5G) with 4 teeth bearing 4 keels in lateral group. Posterior connective lamina of gonapophyses IX with median field with a bat-like prominence (medial dorsal process) (Fig. 5H: b); distal parts of posterior ventral lobes bent under broad angle, olecranon-shaped (Fig. 5H: a).

#### Remarks.

This species was described after the holotype [1 male] from China (Yunnan), deposited in NWAFU.

### Neodurium postfasciatum

Taxon classificationAnimaliaHemipteraIssidae

Fennah, 1956

Figs 1C , 6

Neodurium postfasciatum Fennah, 1956: 513: figs 24E–I; Ran et al. 2005: 570: figs 1–8; Wang and Wang 2011: 552.

#### Material examined.

1♂, China, Hubei, Wudang Mountain, 23 May 23, L.-M. Wang (IEGU); 1♂, Hubei, Houhe National Nature Reserve, 22 July 2013, Z.-M. Chang (IEGU).

#### Male genitalia.

**Figure 6.**
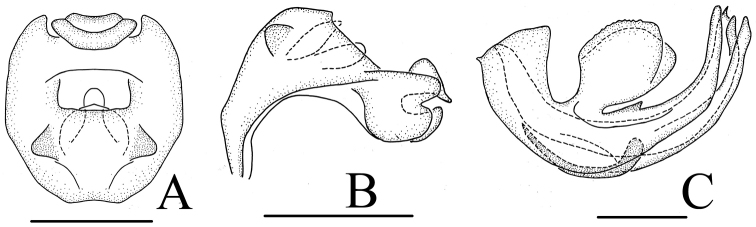
*Neodurium postfasciatum* Fennah, 1956 male. **A** anal segment, dorsal view **B** anal segment, lateral view **C** aedeagus and phallobase, lateral view. Scale bars: 0.5 mm (**A–C**).

Anal tube (Fig. 6A, B) relatively short, subquadrate in dorsal view, with two lobes near basal part, with three lobes in apical margin, median lobe ship-like. Anal column (Fig. 6A, B) short, located at the middle of anal tube. Phallobase (Fig. 6C) with dorsal lobe relatively long, not reaching the tip of lateral lobe, with fan-like process on dorsal margin, with small dentate tooth after fan-like process; lateral lobe split into two branches in lateral view, apical part with sharp tooth. Aedeagus (Fig. 6C) with pair of long hook-like processes in lateral view.

#### Remarks.

This species was firstly described of females from China (Hubei), deposited in CAAS (Fennah 1956). It was subsequently redescribed including the male genitalia by Ran et al. (2005).

### Neodurium weiningensis

Taxon classificationAnimaliaHemipteraIssidae

Zhang & Chen, 2008

Figs 1D , 7

Neodurium weiningensis Zhang & Chen, 2008: 65: figs 1–9.

#### Material examined.

3♂♂, 3♀♀, China, Guizhou, Weining, 20 Aug. 1983, Z.-Z. Li (IEGU); 3♂♂, 3♀♀, Guizhou, Weining, 24 Aug. 2008, Y. Liu (IEGU); 1♂, Guizhou, Weining, 29 Sept. 2005, Q.- R. Liao (IEGU); 1♂, 1♀, Guizhou, Weining, 24 Aug. 2008, Y. Liu (BMNH.)

#### Female genitalia.

**Figure 7.**
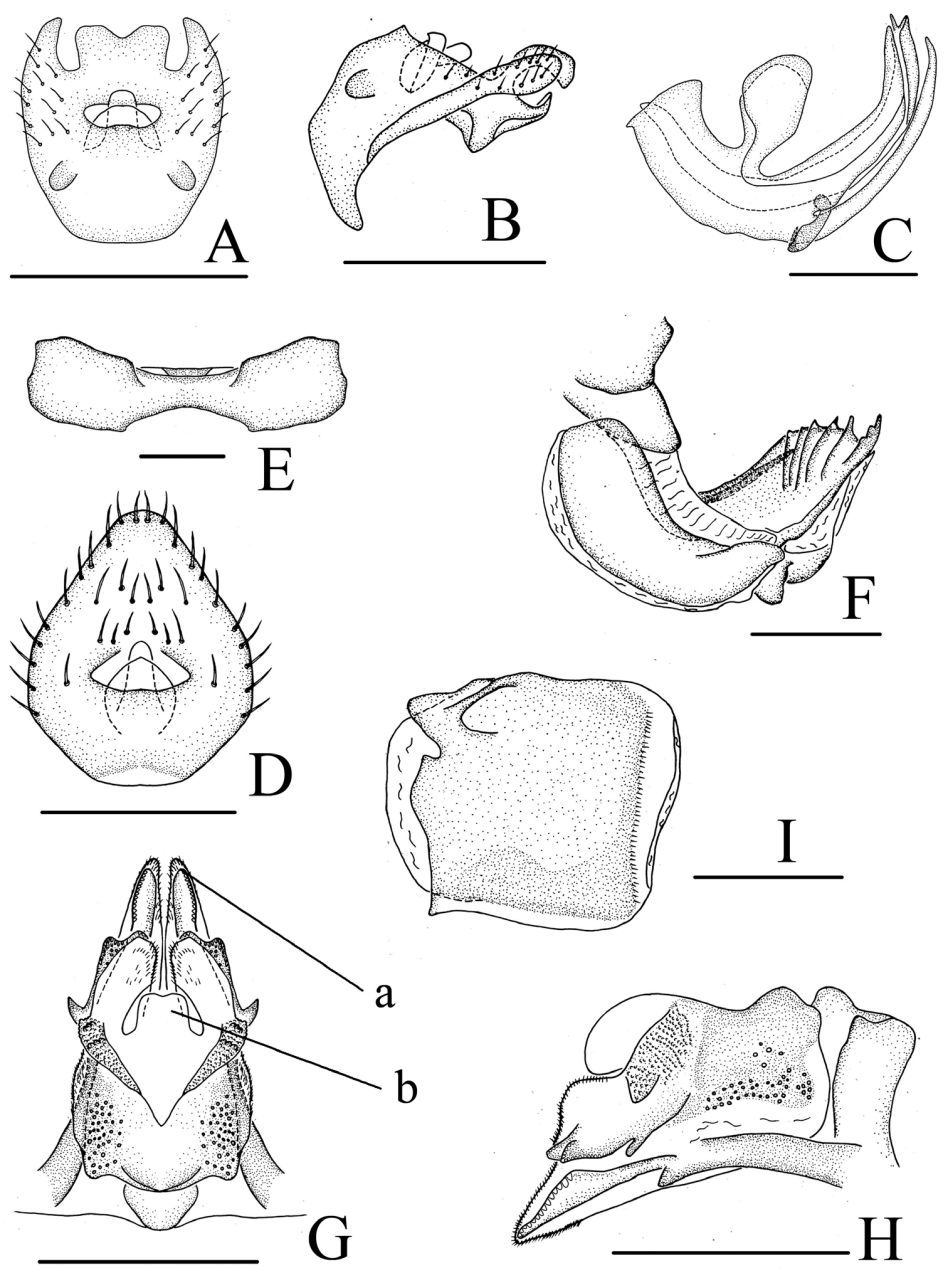
*Neodurium weiningensis* Zhang & Chen, 2008. **A–C** male **A** anal segment, dorsal view **B** anal segment, lateral view **C** aedeagus and phallobase, lateral view **D–I** female **D** anal segment, dorsal view **E** sternum VII, ventral view **F** anterior connective lamina of gonapophyses VIII, lateral view **G** posterior connective lamina of gonapophyses IX, dorsal view **H** posterior connective lamina of gonapophyses IX, lateral view **I** gonoplacs, lateral view. a- posterior ventral lobes, b- process of median field. Scale bars: 0.5 mm (**A–I**).

As in *Neodurium fennahi* but anal tube (Fig. 7D) sub-rhomboid, apical part truncate then broad, the widest breadth at the basal 1/3; anal colum short moderately slender, located the basal 1/3 of anal tube. Anterior connective lamina of gonapophyses VIII (Fig. 7F) with 5 teeth bearing 5 keels in lateral group. Posterior connective lamina of gonapophyses IX with median field with a sub-quadrate prominence (medial dorsal process), apical part wavy (Fig. 7G: b); distal parts of posterior ventral lobes bent at slender angle, blade-like (Fig. 7G: a).

#### Remarks.

This species was described after the holotype and paratypes [three males and four females] from China (Guizhou), deposited in IEGU.

## Discussion

Compared with the structure of male genitalia of this genus, we found that in the structure of the male genitalia *Neodurium hamatum* differs significantly from other species of the genus by the following characters: genital styles without a hook-like process on the dorsal margin in lateral view (Fig. 5D); anal tube without two sub-basal lobes (Fig. 5A), without other lobes on apical margin in dorsal view and without various process ventrally in lateral view (Fig. 5B); anal column long and slender. Possibly *Neodurium hamatum* belongs to another subgenus. On the other side, according to the female genitalia of this genus, *Neodurium hamatum* is distinctly different from other species as follows: Anterior connective lamina of gonapophyses VIII short, relatively broad (Fig. 5G). Posterior connective lamina of gonapophyses IX broad, distal parts of posterior ventral lobes bent under broad angle, olecranon-shaped (Fig. 5H). These characters also show that the above assumption seemed reasonable.

However, how much value does the female genitalia have in Parahiraciini? Hamilton (2011) stated that the ovipositor was an important character in Fulgoroidea and the female genitalia of Issidae have attracted increasing attention in recent years with some important characters being identified by Gnezdilov (2003, 2014). However, little information has been reported for Parahiraciini, except for *Bardunia*, *Flavina*, *Folifemurum*, *Narinosus*, *Scantinius* and *Tetricodes* (Gnezdilov 2011, Zhang et al. 2010, Che et al. 2013, Gnezdilov and Wilson 2005, 2007, Fennah 1956). Although, Che et al. (2013) discussed relationships within Parahiraciini with respect to characters of the vertex, forewings and hind wings, much still remains to be done to using the female genitalia.

## Supplementary Material

XML Treatment for Neodurium

XML Treatment for Neodurium fennahi

XML Treatment for Neodurium digitiformum

XML Treatment for Neodurium duplicadigitum

XML Treatment for Neodurium flatidum

XML Treatment for Neodurium hamatum

XML Treatment for Neodurium postfasciatum

XML Treatment for Neodurium weiningensis
